# Inhibition of mitochondrial complex I by the novel compound FSL0260 enhances high salinity-stress tolerance in *Arabidopsis thaliana*

**DOI:** 10.1038/s41598-020-65614-9

**Published:** 2020-05-26

**Authors:** Kaori Sako, Yushi Futamura, Takeshi Shimizu, Akihiro Matsui, Hiroyuki Hirano, Yasumitsu Kondoh, Makoto Muroi, Harumi Aono, Maho Tanaka, Kaori Honda, Kenshirou Shimizu, Makoto Kawatani, Takeshi Nakano, Hiroyuki Osada, Ko Noguchi, Motoaki Seki

**Affiliations:** 1 Plant Genomic Network Research Team, RIKEN Center for Sustainable Resource Science (CSRS), Yokohama, 230-0045 Japan; 2 Department of Advanced Bioscience, Faculty of Agriculture, Kindai University, Nara, 631-8505 Japan; 3 Chemical Biology Research Group, RIKEN CSRS, Wako, Saitama, 351-0198 Japan; 4 Chemical Resource Development Research Unit, RIKEN CSRS, Wako, Saitama 351-0198 Japan; 5 Gene Discovery Research Group, RIKEN CSRS, Wako, Saitama 351-0198 Japan; 6 Graduate School of Biotsudies, Kyoto University, Kyoto, 606-8502 Japan; 7 School of Life Sciences, Tokyo University of Pharmacy and Life Sciences, Hachioji, Tokyo 192-0392 Japan; 8 Kihara Institute for Biological Research, Yokohama City University, Yokohama, 244-0813 Japan; 9 CREST, JST, Kawaguchi, Saitama, 332-0012 Japan; 10 Plant Epigenome Regulation Laboratory, RIKEN Cluster for Pioneering Research, Wako, Saitama 351-0198 Japan

**Keywords:** Salt, Plant sciences, Abiotic

## Abstract

Chemical priming is an attractive and promising approach to improve abiotic stress tolerance in a broad variety of plant species. We screened the RIKEN Natural Products Depository (NPDepo) chemical library and identified a novel compound, FSL0260, enhancing salinity-stress tolerance in *Arabidopsis thaliana* and rice. Through transcriptome analysis using *A. thaliana* seedlings, treatment of FSL0260 elevated an alternative respiration pathway in mitochondria that modulates accumulation of reactive oxygen species (ROS). From comparison analysis, we realized that the alternative respiration pathway was induced by treatment of known mitochondrial inhibitors. We confirmed that known inhibitors of mitochondrial complex I, such as rotenone and piericidin A, also enhanced salt-stress tolerance in *Arabidopsis*. We demonstrated that FSL0260 binds to complex I of the mitochondrial electron transport chain and inhibits its activity, suggesting that inhibition of mitochondrial complex I activates an alternative respiration pathway resulting in reduction of ROS accumulation and enhancement of tolerance to salinity in plants. Furthermore, FSL0260 preferentially inhibited plant mitochondrial complex I rather than a mammalian complex, implying that FSL0260 has a potential to be an agent for improving salt-stress tolerance in agriculture that is low toxicity to humans.

## Introduction

High salinity stress is one of the major abiotic factors limiting crop productivity. Globally, more than 20% of irrigated lands are affected by soil salinization^1^. Improving crop salinity-stress tolerance is essential for sustainable food production. High salinity stress induces osmotic stress and ionic stress that inhibit water uptake in roots and photosynthesis in shoots^2^. It also enhances the generation of reactive oxygen species (ROS) such as superoxide anion (O_2_^·^^−^), hydrogen peroxide (H_2_O_2_) and the hydroxyl radical (·OH). ROS works as a signaling molecule; however, excess ROS is highly toxic and leads to metabolic disorders, cell damage and cell death^3–5^. Salinity stress causes over-reduction of the mitochondrial electron transport chain (mETC), which induces electron leakage to O_2_ and subsequent production of O_2_^·^^−^ and H_2_O_2_^6^. The complex I and III of the mETC are known as major ROS-producing sites during abiotic stress^5^. In plants, mETC contains an alternative respiration pathway consisting of type II NAD(P)H dehydrogenase (ND) and the alternative oxidase (AOX). The alternative respiration pathway works as a bypass of mETC and does not directly contribute to proton pumping or ATP synthesis, and excess energy is released as heat. The alternative respiration pathway is considered to be important for preventing over-reduction of mETC and for mitigating ROS production^7^. NDs are classified into internal and external NAD(P)H dehydrogenases and reduces ubiquinone bypassing complex I^8^. AOX bypasses the proton-pumping complexes III and IV and acts to maintain the reduction state of the UQ pool and to lower ROS production in mitochondria^9^. In *Arabidopsis*, there are seven type II NDs (NDB1–4, NDA1–2 and NDC1) and five AOXs (AOX1a–d and AOX2). These alternative respiration pathway genes are induced at a gene, protein and activity level by a variety of stress conditions^10^, suggesting that this pathway is important for stress response.

So far, genetic approaches including conventional breeding and gene recombination have been employed as strategies to enhance stress tolerance. However, breeding is time-consuming, and genetically modified crops are unacceptable in many countries around the world. Instead, recent research suggests that the chemical priming may improve abiotic stress tolerance in plants^11^. We previously showed that histone deacetylase inhibitors and ethanol enhanced salinity-stress tolerance in plants including *Arabidopsis*, rice and cassava^12–15^. Those studies indicated that chemical compounds have the potential to improve tolerance in various agricultural crops. We therefore screened the RIKEN Natural Products Depository (NPDepo) chemical library to identify further compounds enhancing salinity-stress tolerance. A novel compound, FSL0260, was identified as enhancing salt-stress tolerance in plants. We demonstrated that FSL0260 works as an inhibitor of mitochondrial complex I and enhances an alternative respiration pathway, resulting in reduction of ROS accumulation and enhancement of salt-stress tolerance.

## Results

### FSL0260 is identified as a novel compound enhancing salt stress tolerance in *A. thaliana*

**Figure 1:**
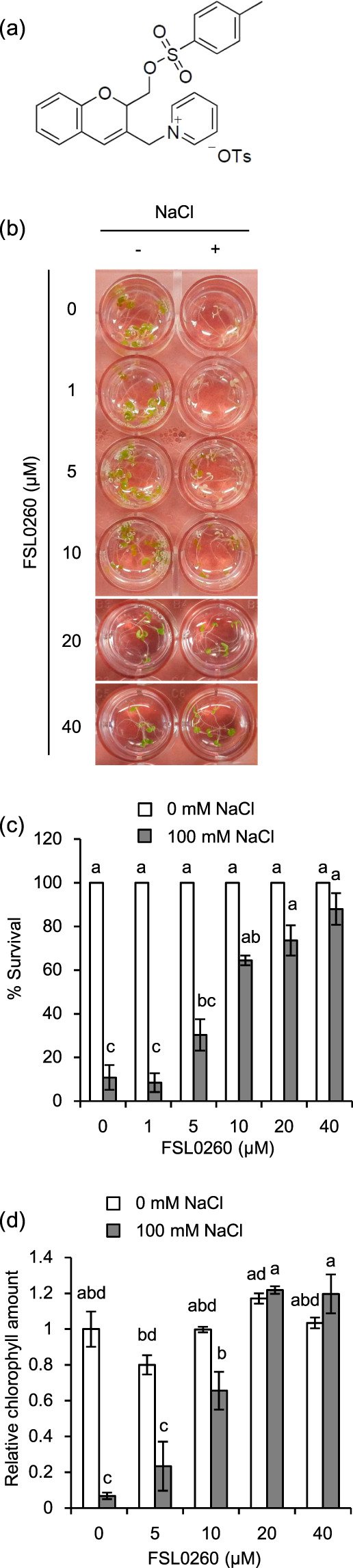
FSL0260 enhances high salinity stress tolerance in *Arabidopsis thaliana*. (**a**) Structure of FSL0260. (**b**) Morphology of seedlings treated with 0–40 µM FSL0260, with or without a subsequent treatment of 100 mM NaCl for 4 days. DMSO was used as negative control. Inside diameter of the well is 15.4 mm. (**c**) Survival rate under high-salinity conditions in the presence or absence of 0–40 µM FSL0260. The survival rate of 15 plants was calculated 4 days after NaCl treatment. (**d**) Chlorophyll content in 0–40 µM FSL0260-treated plants under normal or high-salinity conditions. The chlorophyll content of the plants treated with 0 µM FSL0260 was set as 1. These experiments were conducted using three biological replicates. Error bars represent the mean ± standard error (SE). Statistical significance was determined by ANOVA, followed by post-hoc Tukey’s tests. Means that differed significantly (P < 0.05) are indicated by different letters.

To identify compounds that enhance salt-stress tolerance in plants, we screened 405 compounds from the NPDepo library. *Arabidopsis* wild-type Columbia-0 (Col-0) seeds were grown in liquid culture medium containing each compound for 4 days, and then plants were treated with 100 mM NaCl. We then observed survival rates for 4 days, and identified four compounds that increased survival rates under high salt-stress conditions. Among them, we focused on 2-[[[(4-methylphenyl)sulfonyl]oxy]methyl]-2H-1-benzopyran-3-yl]methylpyridin-1-ium 4-methylbenzenesulfonate (1:1) (FSL0260) (Fig. 1a), because it showed the strongest tolerance to salinity stress. To confirm the salinity-stress tolerance by FSL0260, wild-type plants grown in liquid culture medium for 4 days were treated with 0–40 µM FSL0260 for 24 h, with or without subsequent treatment with 100 mM NaCl for 4 days. The plants treated with FSL0260 increased their survival rate in a dose-dependent manner under salinity-stress conditions (Fig. 1b,c). We observed that the chlorophyll content of plants treated with more than 20 µM FSL0260 under salinity stress was recovered at the same level as that of plants under normal conditions (Fig. 1d), and confirmed that FSL0260 enhanced salinity-stress tolerance. However, high concentrations of FSL0260 treatment inhibited plant growth (Supplementary Fig. S1). As 20 µM FSL0260 greatly enhanced salinity-stress tolerance and minimized growth inhibition, we adopted 20 µM FSL0260 for further analysis. In addition, we confirmed that FSL0260 enhanced salinity-stress tolerance not only in liquid culture but also in solid agar plates (Supplementary Fig. S2a,b).

### Mitochondrial alternative respiration pathway genes are up-regulated by FSL0260 treatment under salinity stress

**Figure 2:**
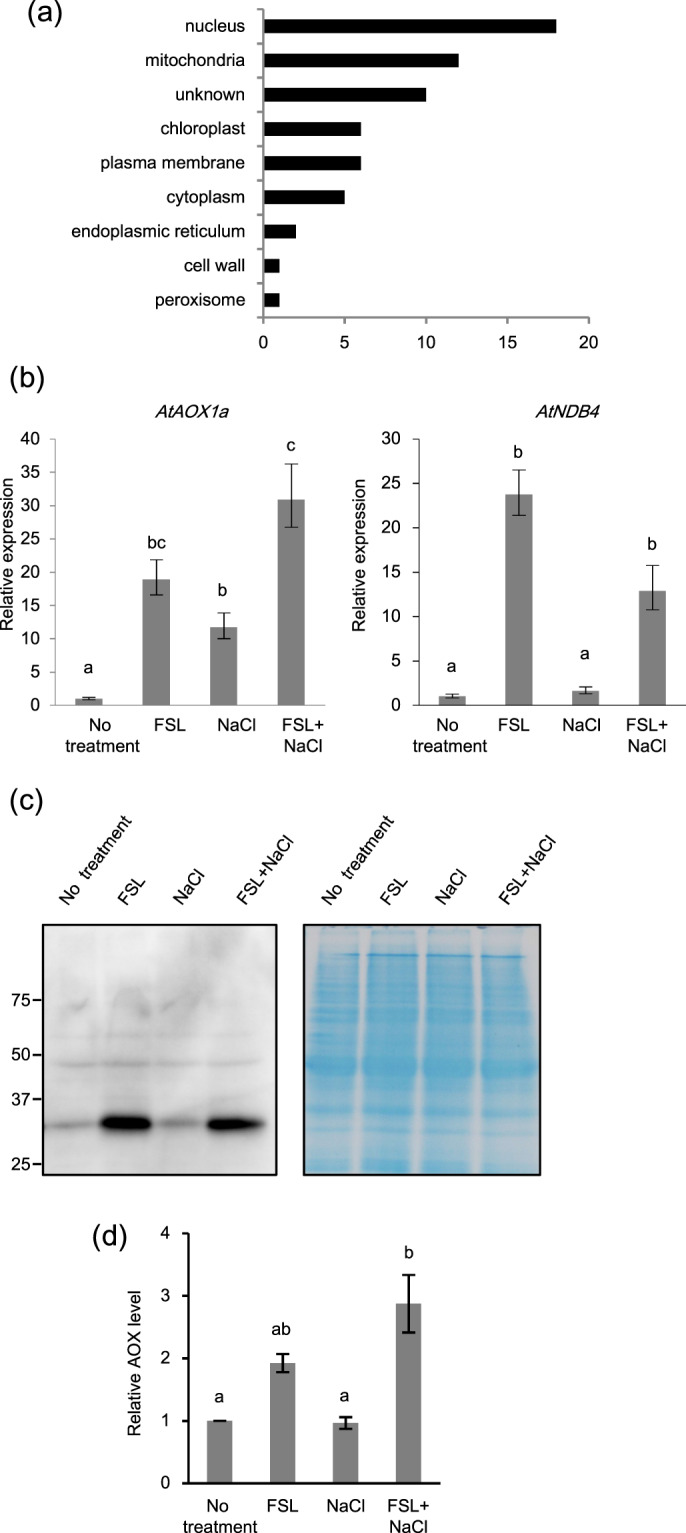
Expression profile of genes up-regulated by both FSL0260 treatment and salinity stress. (**a**) Cellular component gene ontology of up-regulated genes by FSL0260 treatment. (**b**) Relative expression levels of *AtNDB4* and *AtAOX1a* genes during salinity-stress treatment for 0 and 2 h with or without 20 µM FSL0260. Expression level of plants treated with DMSO was set as 1. 18S rRNA was used as an internal standard. Error bars represent the mean ± SE (n = 3). Statistical significance was determined by ANOVA, followed by post-hoc Tukey’s tests. Means that differed significantly (P < 0.05) are indicated by different letters. (**c**) Immunoblot of the AOX (35 kDa) proteins (left). Coomassie blue-stained gel showing control loading (right). Total proteins were extracted from seedlings treated with 0 or 20 µM FSL0260 for 24 h and with or without subsequent treatment of 100 mM NaCl for 6 h. DMSO was used as a negative control. Immunoblot analysis was performed using an anti-AOX1/2 antibody. (d) The signal intensity of AOX1/2. DMSO treatment was taken as 1. Error bars represent the mean ± SE (n = 3). Statistical significance was determined by ANOVA, followed by post-hoc Tukey’s tests. Means that differed significantly (P < 0.05) are indicated by different letters.

To elucidate the molecular mechanism of high salt-stress tolerance mediated by FSL0260, we performed global gene expression analysis with a microarray. Four-day-old plants treated with 20 µM FSL0260 or dimethyl sulfoxide (DMSO) for 24 h were examined. We identified 58 genes whose expression was up-regulated by 24 h FSL0260 treatment (Supplementary Table S1). We found that a large proportion of genes which were annotated as mitochondrial proteins were up-regulated by FSL0260 treatment (Fig. 2a). We focused particularly on mitochondrial alternative respiration pathway genes (*AtAOX1a* and *AtNDB4*), since they have been reported to detoxify ROS^9^. The expression profiles of *AtAOX1a* and *AtNDB4* were confirmed by quantitative real-time PCR (qRT-PCR). The expressions of these genes were up-regulated by FSL0260 treatment (Fig. 2b). Next, we confirmed the protein levels of AOX in plants treated with FSL0260. We used non-reducing SDS-PAGE electrophoresis followed by protein gel blotting and evaluated the AOX protein level. Reduced active form AOX (about 35 kDa) was increased by FSL0260 treatment and by both FSL0260 and NaCl treatments (Fig. 2c,d), consistent with the transcription level of *AtAOX1a* under FSL0260 treatment. These results suggest that the salt tolerance conferred by FSL0260 might be due to promotion of ROS detoxification.

### Mitochondrial complex I inhibitor enhances salinity-stress tolerance in *A*. *thaliana*

**Figure 3:**
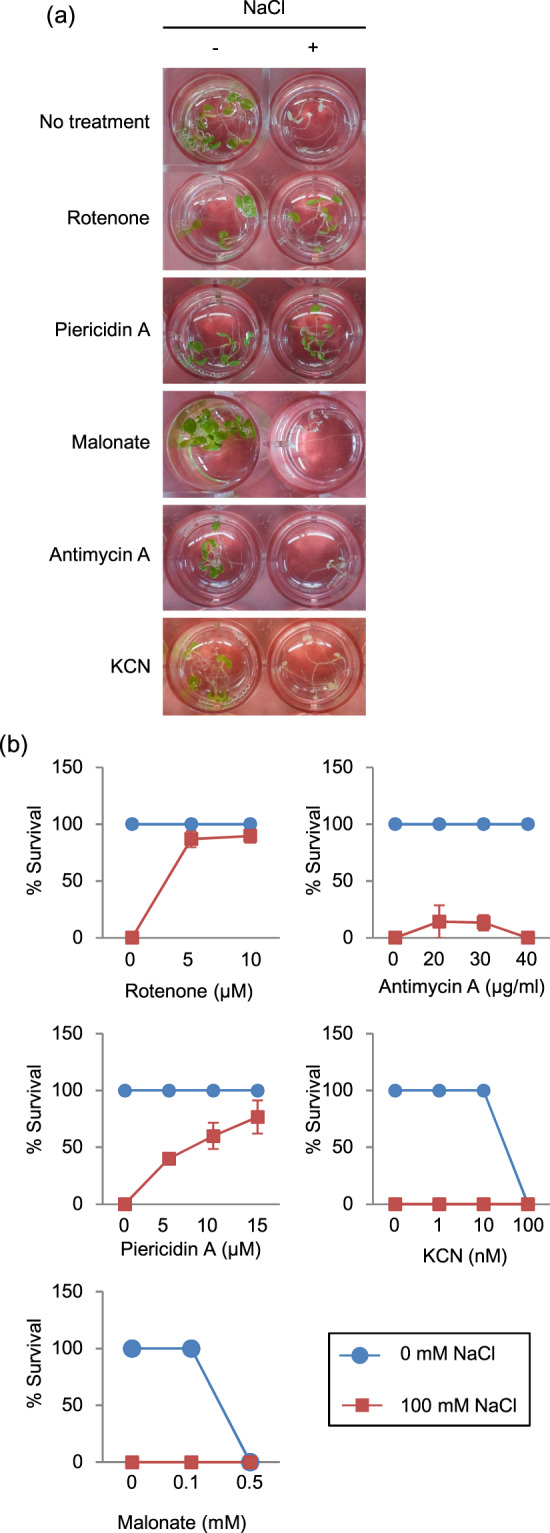
Inhibitors of mitochondrial complex I enhance high salinity stress tolerance. (**a**) Morphology of seedlings treated with 5 µM rotenone, 15 µM piericidin A, 0.1 mM malonate 40 µg/mL antimycin A (AA) and 10 nM KCN with or without subsequent treatment with 100 mM NaCl for 4 days. DMSO was used as negative control. Inside diameter of the well is 15.4 mm. (**b**) Survival rate of plants treated with various mitochondrial inhibitors under high-salinity conditions. The survival rate of 15 plants was calculated 4 days after NaCl treatment. Lines with circles and squares designate the survival rates of inhibitor-treated plants under normal and salt-stress growth conditions, respectively. The experiment was conducted using three biological replicates. Error bars represent the mean ± SE.

Previous transcriptome analysis showed that the treatment of inhibitors of the electron transport chain in mitochondria induced alternative respiration pathway genes^16,17^. Our transcriptome analysis showed that alternative respiration pathway genes were up-regulated by FSL0260 treatment, thus we speculated that FSL0260 works as a mitochondrial inhibitor. To verify this hypothesis, we first examined whether mitochondrial inhibitors affected salinity-stress responses. Four-day-old plants were treated with rotenone and piericidin A (which are complex I inhibitors), malonate (complex II inhibitor), antimycin A (AA) (complex III inhibitor), and KCN (complex IV inhibitor) for 24 h, respectively. They were subsequently treated with NaCl for 4 days and their survival rate was checked. Plants treated with rotenone or piericidin A were able to survive in a dose-dependent manner under high salinity stress; however, malonate, AA and KCN were unable to rescue plants under high salt conditions (Fig. 3a,b). We confirmed that rotenone treatment, as well as the FSL0260 treatment, increased gene expressions of *AtAOX1a* and *AtNDB4* (Supplementary Fig. S3), suggesting that the inhibition of complex I enhances salt-stress tolerance and that FSL0260 is also an inhibitor of mitochondrial complex I.

### FSL0260 binds to mitochondrial complex I and inhibits its activity in plants

**Figure 4:**
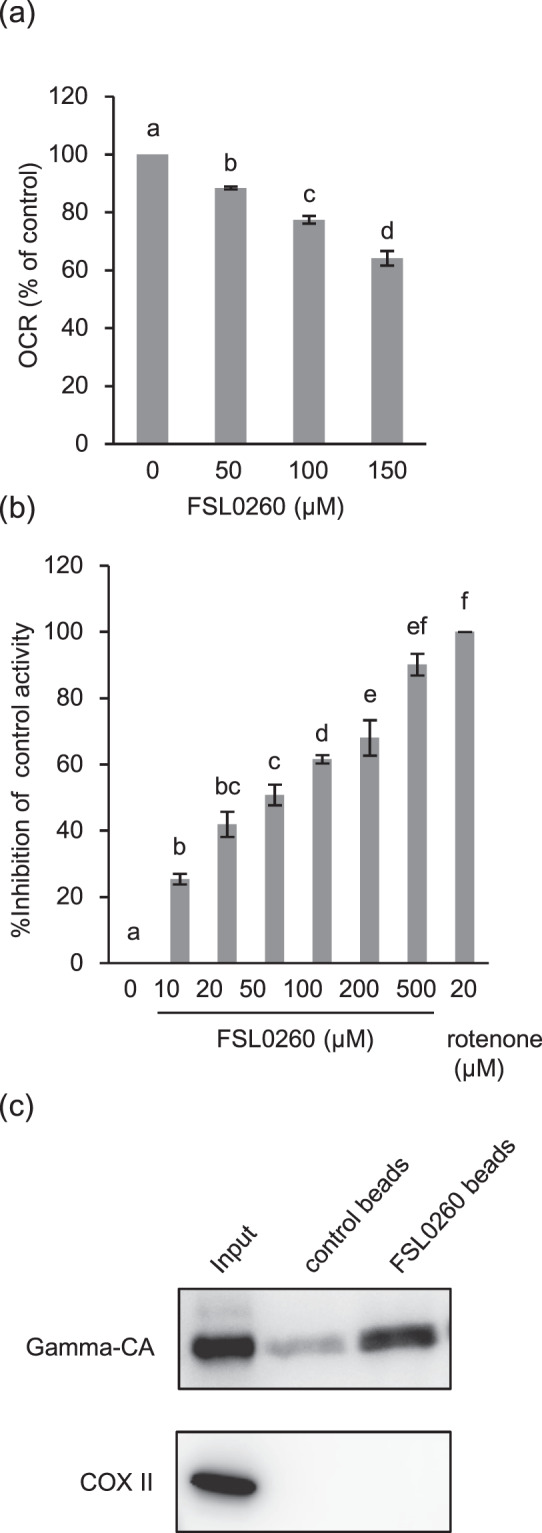
FSL0260 interacts with mitochondrial complex I and inhibits its activity. (**a**) Inhibition of complex I by FSL0260. Oxygen consumption rate (OCR) of isolated mitochondria from potato tuber was monitored with deamino-NADH in the absence or presence of FSL0260. (**b**) Inhibition of deamino-NADH oxidation by FSL0260. Deamino-NADH oxidation was measured by spectrometry using sonicated mitochondria isolated from the potato tuber. The experiment was conducted using three biological replicates. Error bars represent the mean ± SE (n = 3). (**c**) Pull-down assay of FLS0260. Sonicated potato mitochondria were incubated with control or FSL0260 beads. Immunoblot assay was performed using an anti-gamma CA antibody and an anti-COX II antibody.

To investigate whether FSL0260 inhibits mitochondrial complex I activity, we isolated mitochondria from potato tubers and measured complex I activity in two ways: (1) oxygen consumption rate (OCR) was measured as an index for oxidation of deamino-NADH, which is an NADH analog and specific substrate of complex I^18^. FSL0260 treatment inhibits oxidation of deamino-NADH in a dose-dependent manner (Fig. 4a); and (2) we assessed oxidation of deamino-NADH by spectrometry. Results showed that FSL0260 treatment decreased oxidation of deamino-NADH in a concentration-dependent manner (Fig. 4b), suggesting that FSL0260 has inhibition activity in respect of mitochondrial complex I (IC50 = 45.1 µM). Furthermore, we measured OCR for assessing activity of complex IV and activity of complex II by spectrometry. These results confirmed that FSL0260 did not inhibit complex II and IV (Supplementary Fig. S4a,b). We also assessed whether FSL0260 worked in mammalian mitochondria, and found that it did not inhibit mitochondria isolated from the bovine heart (Supplementary Fig. S5), suggesting that FSL0260 preferably inhibits plant mitochondria.

To confirm whether FSL0260 binds to mitochondrial complex I, a pull-down assay was performed using FSL0260-fixed agarose beads. Gamma-CA, a subunit of mitochondrial complex I, was significantly pulled down with FSL0260-bound beads. Control beads bound only slightly with gamma-CA (Fig. 4c). COX II, a subunit of mitochondrial complex IV, was not pulled down with both control beads and FSL0260-bound beads (Fig. 4c), indicating the physical interaction of FSL0260 and mitochondrial complex I. These results revealed that FSL0260 is a novel inhibitor of mitochondrial complex I in plants.

### FSL0260 enhances the detoxification of ROS under high salinity-stress conditions

**Figure 5:**
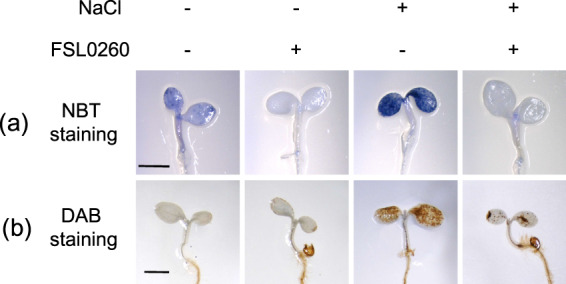
ROS accumulation is reduced by FSL0260 treatment. NBT and DAB staining were used to assess the accumulation of O_2_•− and H_2_O_2_, respectively. *Arabidopsis thaliana* plants were treated with NaCl for 6 h in the presence or absence of 20 µM FSL0260. Bar = 1 mm. Each treatment was analyzed using 10 plants. Three biological repeats were performed.

The accumulation of O_2_^•−^ and H_2_O_2_, which are the two main ROS components induced by salt stress, normally results in oxidative damage. We investigated the accumulation of O_2_^•−^ and H_2_O_2_ in FSL0260-treated plants under salt-stress conditions using NBT to detect O_2_^•−^ and DAB staining for monitoring H_2_O_2_. The cotyledons of NaCl-treated plants were significantly stained by NBT and DAB, indicating that O_2_^•−^ or H_2_O_2_ were highly accumulated under salinity-stress conditions. NBT staining showed that FSL0260 treatment led to a greatly reduced accumulation of O_2_^•−^ in plants (Fig. 5a). DAB staining also showed decreased accumulation of H_2_O_2_ by FSL0260 treatment (Fig. 5b). Some chemical compounds – such as ascorbic acid – have radical scavenging activity, thus we confirmed that FSL0260 itself did not show antioxidant activity (Supplementary Fig. S6a,b). FSL0260 treatment induced AOX, thus we investigated ROS level of plants treated with both FSL0260 and salicylhydroxamic acid (SHAM) which is an inhibitor of AOX. The reduction of O_2_^•−^ by FSL0260 under salt stress was repressed by SHAM (Supplementary Fig. S7). These data suggested that up-regulation of alternative respiration pathway by FSL0260 detoxifies ROS and enhances salinity-stress tolerance in *A. thaliana*.

### FSL0260 treatment enhances high-salinity tolerance by decreasing the accumulation of ROS in rice

**Figure 6:**
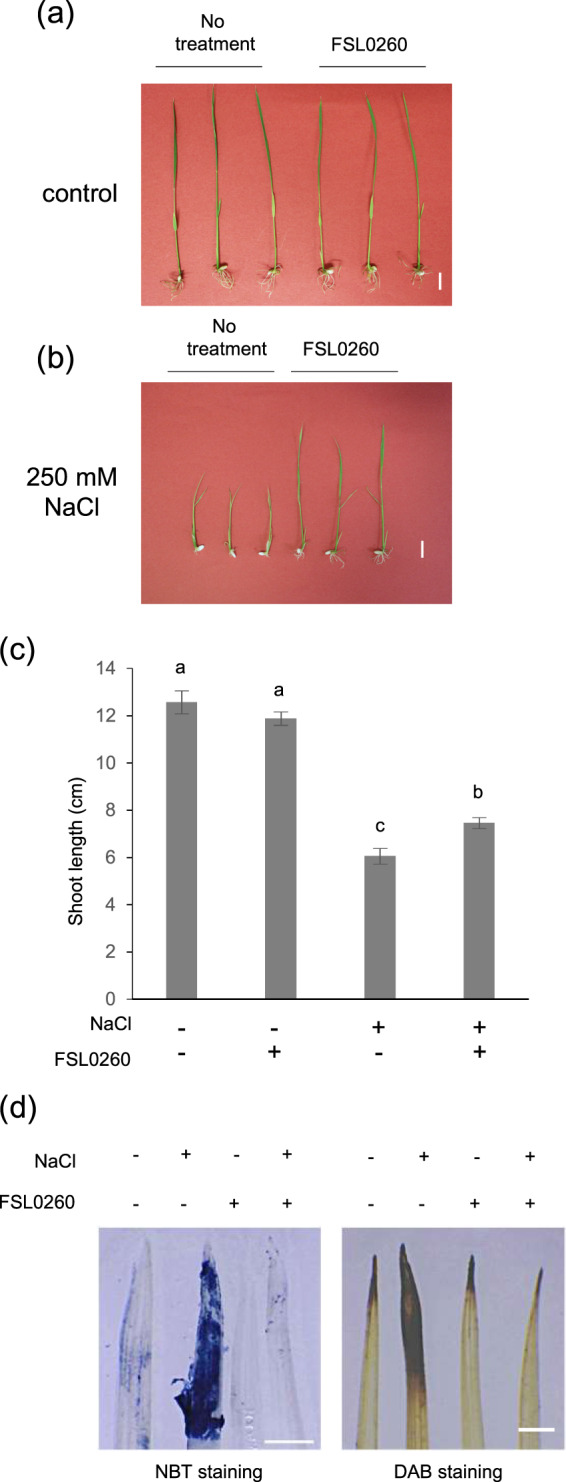
FSL0260 enhances high salinity stress tolerance in *Oryza sativa*. Morphology of rice seedlings without NaCl (**a**), and treated with 250 mM NaCl for 7 days in the presence or absence of 20 µM FSL0260 (**b**). DMSO was used as negative control. Bar = 1 cm. (**c**) Shoot length of rice seedlings under normal condition and salt stress. (d) NBT and DAB staining were used to assess the accumulation of O_2_^•−^ and H_2_O_2_, respectively. 7-day-old rice were treated with NaCl for 24 h in the presence or absence of 20 µM FSL0260. Bar = 1 mm. Three biological repeats were performed.

To confirm whether FSL0260 enhances the tolerance of monocots to salt stress condition, 8-day-old rice seedlings were treated with FSL0260 for 2 days, and then plants were treated with 250 mM NaCl for 7 days. Under normal condition, no significant morphological differences were observed between control plants and FSL0260 treated plants (Fig. 6a). On the other hand, FSL0260 treated plants showed increased shoot growth compared to non-treated plants under salt stress condition (Fig. 6b,c), suggesting that FSL0260 enhances salinity stress tolerance in rice as well as *A. thaliana*.

FSL0260 treatments enhanced ROS detoxification and improved the salt stress tolerance of *A. thaliana* plants. We verified that salinity stress tolerance in rice is also due to the detoxification of ROS by NBT and DAB staining. Rice leaves were more extensively stained by NBT and DAB under salinity stress condition than under control condition (Fig. 6d). However, the intensity of the NBT and DAB staining decreased in FSL0260 treated plants under salt stress condition, suggesting that FSL0260 inhibited ROS accumulation (Fig. 6d), implying that FSL0260 increases salinity tolerance in rice by inhibiting ROS accumulation, similar to its effects in *A. thaliana*.

## Discussion

In this study, we identified a novel compound FSL0260 enhancing salinity-stress tolerance from the chemical screening. The results suggest that FSL0260 has inhibitory activity of mitochondrial complex I and this inhibition induces an alternative respiration pathway including *AtAOX1a* and *AtNDB4*. Up-regulation of an alternative respiration pathway by FSL0260 treatment is associated with reduction in ROS accumulation, leading to enhanced salinity-stress tolerance in plants.

Our results are consistent with a previous report showing that *Arabidopsis* plants overexpressing *AtAOX1a* genes exhibited increased salt-stress tolerance following lower ROS levels under salinity stress^19^. The RNAi line of *AtNDB4* showed reduction in ROS accumulation and salt-stress tolerance caused by enhancing *AtNDB2* and *AtAOX1a*^20^, and plant overexpressing both *AtNDB2* and *AtAOX1a* showed increased NADH oxidation rates and enhanced tolerance to drought and high-light stress^21^, suggesting that there is functional link between AtNDB and AtAOX and total amount of alternative respiration pathway-related genes affects ROS detoxifying activity. Thus, upregulation of both *AtNDB4* and *AtAOX1a* by FSL0260 treatment probably contributes to decrease ROS accumulation and enhances salt stress tolerance.

Our microarray data indicated that the expression of some mitochondrial stress responsive genes was upregulated by FSL0260 treatment. Previous report showed that the expression of *AT12CYS-2* was induced by inactivation of mitochondrial complex I^22^. AtNAC013 and AtNAC044, NAC family transcription factors, have been demonstrated to play crucial roles in mitochondrial retrograde signaling^23,24^. AtNAC013 induces transcription of *AtAOX1a* and *AT12CYS-2* under mitochondrial stress, and *Arabidopsis* plants overexpressing *AtNAC013* genes exhibited increased oxidative-stress tolerance following lower ROS levels under salinity stress^24^. These data provided support the observation that FSL0260 inhibits mitochondrial complex I activity and induces the alternative respiration pathway.

Mitochondrial complex I in *Arabidopsis* is composed of at least 49 subunits, 40 of which represent homologs of bovine complex I and 9 of which are unique to plants^25,26^. In rice, 33 subunits were classified as the component of complex I^27,28^ and they showed the high conservation with that in *Arabidopsis thaliana*^29^. Some deficient mutants of complex I have been reported to improve abiotic stress tolerance in plants. CMSII mutant that has deletion of the *NAD7* in tobacco (*Nicotiana sylvestris*) was found to be more resistant to ozone stress^30^. And *bir6* mutant which is abnormal splicing of NAD7 subunit, enhanced salt and osmotic stress tolerance in *Arabidopsis*^31^. The *ndufs4* mutant (the T-DNA insertion mutant of NDUFS4-encoded 18 kDa Fe-S protein of complex I in *Arabidopsis*) showed enhanced tolerance to cold, mild salt and osmotic stress^32^. Although ROS levels under stress conditions have not been mentioned in these mutants, transcription of the alternative respiration pathway was up-regulated in the CMSII, *ndufs4* and *bir6* mutants^33^. In addition, a mutant of *OsNDUFA9* encoding a mitochondrial complex I subunit in rice was embryo lethal but *OsAOX1a* and *OsAOX1b* were upregulated in the mutant^34^. These observations support our conclusion that inhibition of mitochondrial complex I by FSL0260 enhances an alternative respiration pathway and detoxifies ROS under salinity stress.

Previous reports described that AA treatment induced the expression of *AtAOX1a* and *AtNDB4*^22,35^. However, our result showed that AA treatment did not rescue plants from salinity stress. To investigate cause of this discrepancy, we analyzed ROS accumulation in plants treated with AA. The accumulations of O_2_^•−^ were not significantly different between plants treated with and without AA under salt stress condition (Supplementary Fig. 8), indicating that AA could not repress ROS production even though alternative respiration pathway was activated. We speculated that AOX activation could not fully compensate the inhibition of complex III by AA, then ROS was accumulated in plants treated with AA. The *ppr40-1* knockout mutant which impaired electron transport through complex III, enhanced ROS accumulation and showed the increased sensitivity to salt stress, although gene expression of AOX was upregulated in this mutant^36^, supporting our speculation.

In addition to FSL0260, rotenone and piericidin A also enhanced salinity-stress tolerance in plants. Compared with rotenone, the results suggested that FSL0260 preferentially inhibits plant mitochondrial complex I due to its binding site. Rotenone has been reported to bind to the quinone-binding pocket formed by the PSST, ND1 and the 49-kDa subunit, which are conserved in both mammals and plants^37,38^. It is possible that FSL0260 binds to plant-specific subunits of mitochondrial complex I, resulting in its preference. Fenpyroximate, a mitochondrial complex I inhibitor, displays a selective toxicity to mites and was marketed as an acaricide in the 1990s^39^. This selectivity was caused by rapid hydrolysis in mammals compared with mites^40,41^, suggesting that selectivity in species has advantages in agricultural applications. Although a toxicity test using experimental animals is required to confirm the selectivity of FSL0260, we may expect that FSL0260 could be used as a vitalizing agent in agriculture to enhance salinity-stress tolerance without toxicity to humans.

## Methods

### Plant materials and growth conditions

*A. thaliana* (ecotype Columbia-0) seeds were sterilized and sown in half-strength Murashige and Skoog (MS) liquid medium supplemented with 1% sucrose and 0.1% agar. The plants were grown under previously described conditions^15^. Four-day-old plants were treated with compounds for 24 h, with or without subsequent treatment with 100 mM NaCl (WAKO, Japan). The NaCl solution was added into the medium containing both compound and plants. The survival rate of 20 plants was calculated 4 days after the NaCl treatment. The experiment was conducted using three biological replicates.

For rice experiments, *Oryza sativa* L. cv. Nipponbare seeds were germinated in water at 28 ^◦^C for 3 days, and then transferred to 50 ml tube containing 1/2 MS liquid medium and growth (16-h-light/8-h-dark cycle at 22 °C). For salt stress test, 8-day-old plants were treated with FSL0260 and incubated for 2 days with or without subsequent treatment with 250 mM NaCl for 7 days. Seedling length was measured using ImageJ software (https://imagej.nih.gov/ij/index.html). The data was analyzed from 12 plants for each treatment. The experiment was conducted using two independent biological replicates.

### Chemical materials

The chemical library (10 mg mL^−1^ of each compound in dimethylsulfoxide (DMSO, 5 µL)) was obtained from the RIKEN NPDepo (http://www.cbrg.riken.jp/npedia/?LANG=en). The synthesis of FSL0260 is described in Supplemental methods. Rotenone and Antimycin A (AA) were purchased from Sigma-Aldrich (St. Louis, USA). KCN and Malonate were purchased from WAKO (Osaka, Japan). Piericidin A was purchased from BioAustralis (Smithfield, Australia). Salicylhydroxamic acid was purchased from Tokyo Chemical Industry Co., Ltd. (Tokyo, Japan).

### Chemical library screening

We sowed 5–6 Col-0 seeds in each well containing 500 µL of half-strength MS liquid medium containing 10 µg mL^−1^ chemical in a 48-well plate. Seedlings were grown for 5 days and 100 mM NaCl was added. The survival rates were checked 4 days after the NaCl treatment.

### Measurement of chlorophyll content

Four-day-old *A. thaliana* plants were treated with 0–40 µM FSL0260 for 24 h and then exposed to 100 mM NaCl for 72 h. We then measured the chlorophyll content of 30–50 mg of seedlings for each treatment as previously described^15^. The experiment was conducted with three biological replicates. Statistical significance was determined by ANOVA, followed by post-hoc Tukey’s tests. Means that differed significantly (P < 0.05) are indicated by different letters.

### RNA extraction

Total RNA was extracted from 5-day-old *A. thaliana* seedlings treated with 20 µM FSL0260 for 24 h, with or without subsequent treatment with 100 mM NaCl for 2 h. DMSO was used as a negative control. For qRT-PCR, total RNA was extracted from 5–10 plants using the Plant RNA reagent (Thermo Fisher Scientific, Waltham, USA) as previously described^42^. For microarray analysis, total RNA was extracted from 20 plants with an RNeasy Plant Mini Kit (Qiagen, Hilden Germany) according to the manufacturer’s instructions. The quality of the extracted total RNA was evaluated using a Bioanalyzer system (Agilent, Santa Clara, USA). The experiment was conducted using three biological replicates.

### Microarray analysis

A microarray analysis was completed as previously described^42^. The microarray data were deposited in the GEO database (GEO ID: GSE128820). Each treatment was analyzed using three biological replicates, and a total of ten plants was sampled for each treatment and/or repeat. Genes with an expression log2 ratio ≥ 1 [t test analysis, Benjamini–Hochberg correction (FDR) < 0.05] were identified as up-regulated genes. Gene Ontology assignments for *Arabidopsis* genes were obtained from TAIR (www.arabidopsis.org).

### Quantitative real-time PCR analysis

First-strand cDNA synthesis was performed with a PrimeScript™ RT reagent Kit (Takara, Kusatsu Japan) for quantitative real-time polymerase chain reaction (qRT-PCR) analysis. The qRT-PCR was conducted as previously described^15^ and 18S rRNA was used as a reference gene. The experiment was conducted using three biological replicates, and a total of ten plants was sampled for each treatment and/or repeat. The qRT-PCR primer sequences are shown in Table S2.

### Protein extraction and immunoblot analysis

Seedlings were pulverized in liquid nitrogen with a multi beads shocker (Yasui Kikai, Osaka, Japan), and an SDS sample buffer (without β-mercaptoethanol) was added. After centrifugation at 15,000 rpm for 5 min at 4 °C, the supernatants were transferred into clean tubes. Total proteins were separated onto 12.5% (w/v) acrylamide gels by non-reducing SDS-PAGE. Immunoblotting was performed as described previously^43^ using anti-AOX1/2 (AS04 054), anti-Gamma CA (AS17 4114) and anti-COX II (AS04 053A) (Agrisera, Vännäs, Sweden).

### Isolation of mitochondria

Potato tubers (*Solanum tuberosum* L.) were purchased from local markets. Isolation of intact mitochondria from potato tubers was performed according to Yoshida *et al*.^44^.

### Measurement of oxygen uptake rate by isolated mitochondria

Oxygen uptake rates were measured polarographically with a Clark-type oxygen electrode (Rank Brothers, Cambridge, UK) at 25 °C in 1 mL of air-saturated medium containing 0.3 M sucrose, 10 mM KH_2_PO_4_, 10 mM TES and 10 mM MgCl_2_ (pH 6.8). NADH:ubiquinone oxidoreductase activity was measured for 40 µg of mitochondria which were freeze–thawed three times in the presence of 1 mM deamino-NADH (Sigma-Aldrich, St. Louis, USA). Percentage FSL0260 resistance was calculated using the formula in Eq. (1):

1 $$ \% {\rm{FSL0260}}\,{\rm{resistance}}=\{[{\rm{Rate}}+{\rm{FSL0260}}]/[{\rm{Rate}}-{\rm{FSL}}0260]\}\times 100 \% $$

where Rate is the rate of oxygen consumption (nmol oxygen min^−1^ mg^−1^ of protein).

### Measurement of enzymatic activity

Complex I activity was measured by a modified version of a published method^45,46^. Mitochondria were suspended in 0.35 M mannitol, 0.25 M sucrose and 25 mM TES (pH 7.8). The suspension was sonicated for 6 × 5 s (maximum output), and the debris was removed by centrifugation for 10 min at 16,000 g. The supernatant was used for the experiments. The rate of deamino-NADH oxidation by 0.7 µg sonicated mitochondria was measured spectrophotometrically at 340 nm in a medium containing 50 mM potassium buffer (pH7.5), 1 mM EDTA, 2 mM KCN and 25 µM ubiquinone at 30 °C. The reaction was started by adding 0.2 mM deamino-NADH.

### Pull-down assay

FSL0260 beads were prepared as previously described^47^. We incubated 150 µg sonicated potato mitochondria with FSL0260 beads in 1 mL binding buffer containing 50 mM Tris HCl (pH 7.5), 150 mM NaCl, 0.5% Tween 20, 0.1 mM EDTA and Protease Inhibitor Cocktail for plant cells (Sigma-Aldrich St. Louis, USA) for 3 h at 4 °C. The beads were then washed three times in 1 mL binding buffer and eluted with an SDS sample buffer. The eluted protein was then subjected to SDS-PAGE.

### Staining to detect superoxide anion and hydrogen peroxide

Five-day-old *A. thaliana* plants treated with 20 µM FSL0260 for 24 h, with or without a subsequent treatment with 100 mM NaCl for 6 h and 5 h, were stained for NBT and DAB. For the rice experiments, 8-day-old *O. sativa* L. cv. Nipponbare plants treated with 20 µM FSL0260 for 24 h, with or without a subsequent treatment with 200 mM NaCl for 24 h, were stained for NBT and DAB. NBT and DAB staining was conducted as previously described^13^.

## Supplementary information

Supplementary information and .
