# Supplementary material for: Inhibition of mitochondrial complex I by the novel compound FSL0260 enhances high salinity-stress tolerance in Arabidopsis thaliana
Source: Sci Rep. 2020 May 26;10:8691. doi: 10.1038/s41598-020-65614-9 (PMC7250896; doi:10.1038/s41598-020-65614-9)
Supplement: Supplementary file 1 — Supplementary information and . [file 41598_2020_65614_MOESM1_ESM.pdf]

Inhibition of mitochondrial complex I by the novel compound FSL0260 enhances high salinity-stress tolerance in *Arabidopsis thaliana*

Kaori Sako[1,2,9]*, Yushi Futamura[3], Takeshi Shimizu[3], Akihiro Matsui[1,10], Hiroyuki Hirano[4] Yasumitsu Kondoh[3], Makoto Muroi[3], Harumi Aono[3], Maho Tanaka[1], Kaori Honda[3], Kenshirou Shimizu[3], Makoto Kawatani[3], Takeshi Nakano[5,6], Hiroyuki Osada[3,4], Ko Noguchi[7], Motoaki Seki[1,8,9,10]*

[1]Plant Genomic Network Research Team, RIKEN Center for Sustainable Resource Science (CSRS), Yokohama 230-0045, Japan
[2]Department of Advanced Bioscience, Faculty of Agriculture, Kindai University, Nara, 631-8505,
[3]Chemical Biology Research Group, RIKEN CSRS, Wako, Saitama 351-0198, Japan
[4]Chemical Resource Development Research Unit, RIKEN CSRS, Wako, Saitama 351-0198, Japan
[5]Gene Discovery Research Group, RIKEN CSRS, Wako, Saitama 351-0198, Japan
[6]Graduate School of Biotsudies, Kyoto University, Kitashirakawa, Sakyo, Kyoto 606-8502
[7]School of Life Sciences, Tokyo University of Pharmacy and Life Sciences, Hachioji, Tokyo 192-0392, Japan
[8]Kihara Institute for Biological Research, Yokohama City University, Yokohama 244-0813, Japan
[9]CREST, JST, Kawaguchi, Saitama 332-0012 Japan
[10]Plant Epigenome Regulation Laboratory, RIKEN Cluster for Pioneering Research, Wako, Saitama 351-0198, Japan

***corresponding authors:** Motoaki Seki motoaki.seki@riken.jp,
Kaori Sako kaori.sako@riken.jp

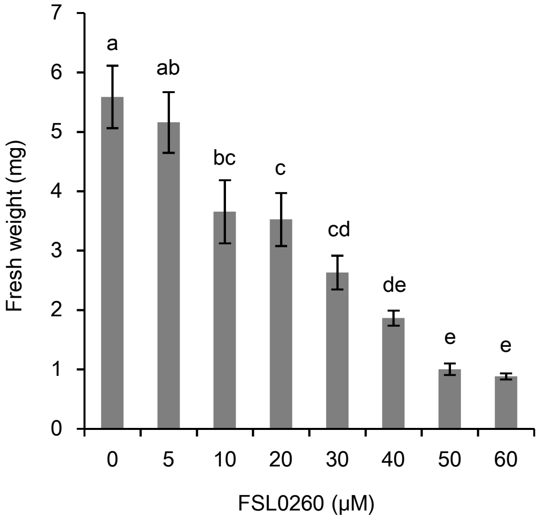

Supplementary figure 1. Fresh weight of plant treated with FSL0260.
Plants were grown for 4 days and treated with 0-60 μM for 4 days, and then measured fresh weight. Error bars represent the mean ± standard error (SE). Statistical significance was determined by ANOVA, followed by post-hoc Tukey's tests. Means that differed significantly ($P<0.05$) are indicated by different.

(a)

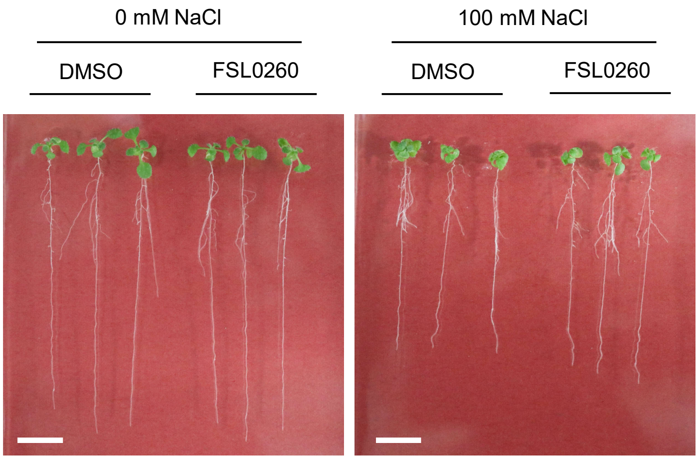

(b)

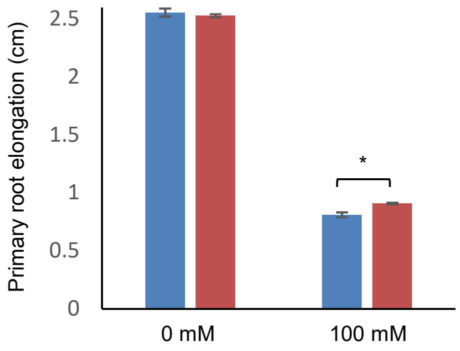

Supplementary figure 2. Root elongations of plant treated with FSL0260 under salinity stress.
Plants were grown for 7 days in MS plates and transferred to MS plates containing 20 μM FSL0260 for 2 days. Then plants were transferred MS plates containing 0 and 100 mM NaCl for 3 days. (A) Morphology of seedlings treated with 20 μM FSL0260 with or without subsequent treatment with 100 mM NaCl for 3 days. DMSO was used as negative control. Bar = 1 cm. (B) Root elongation for 3 days were measured (n=15). Error bars represent the mean ± standard error (SE). Significance was determined according to Student’s t-test. *P < 0.05

Sako et al., Supplementary figure 2

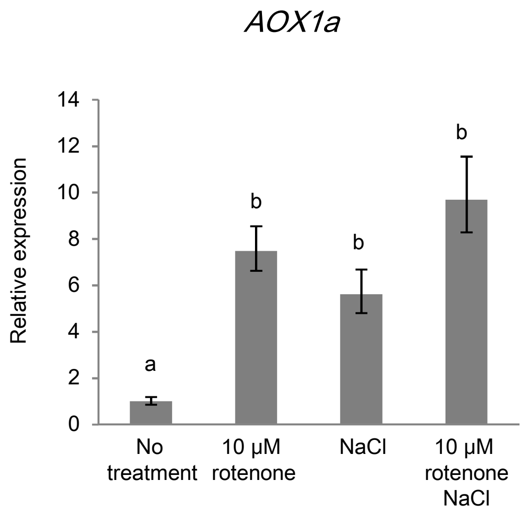

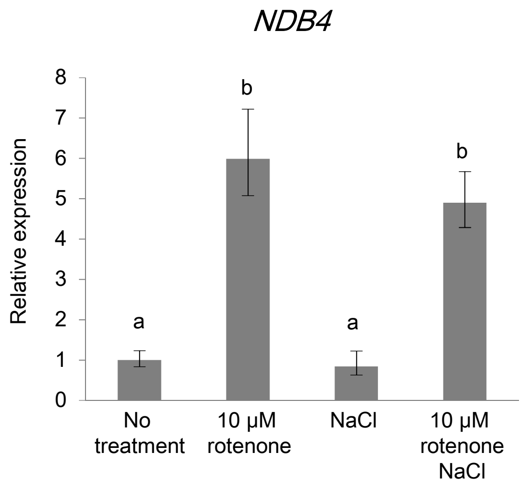

Supplementary figure 3. Gene expression analysis of plants treated with rotenone.
Relative expression levels of the AtAOX1a and AtNDB4 genes during salinity stress treatment for 0 and 2 h with or without 10 μM rotenone. The expression level of the plants treated with DMSO was set as 1. 18S rRNA was used as an internal standard. Error bars represent the means ± SE (n = 3). Statistical significance was determined by ANOVA, followed by post-hoc Tukey's tests. Means that differed significantly ($P < 0.05$) are indicated by different letters.

Sako et al., Supplementary figure 3

(a)

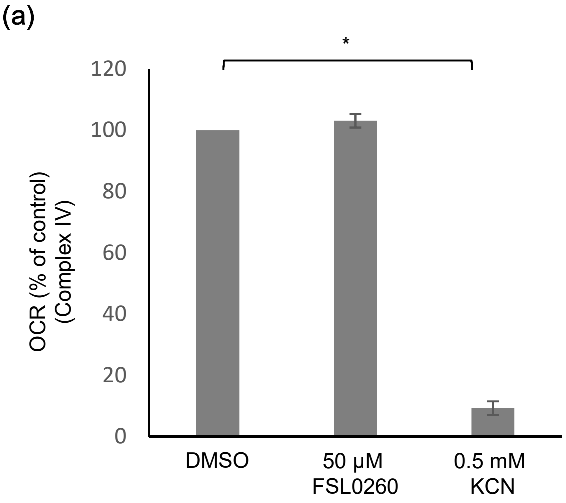

(b)

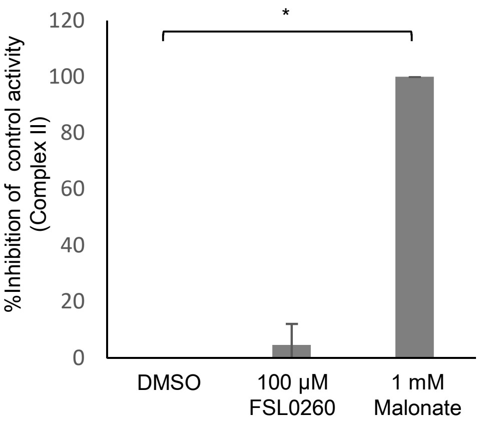

Supplementary figure 4. Activity of mitochondrial complex II and IV in mitochondria treated with FSL0260.
(a) Measurement of activity of complex IV. Oxygen consumption rate (OCR) of isolated mitochondria from potato tuber was monitored with cytochrome c in the absence or presence of FSL0260. (b) Activity of succinate dehydrogenase (Complex II). The reduction of 2,6-Dichloroindophenol Sodium Salt Dihydrate (DCPIP) was measured by spectrometry using isolated mitochondria with or without FSL0260. Error bars represent the mean ± SE (n = 3). Significance was determined according to Student’s t-test. *P < 0.05.

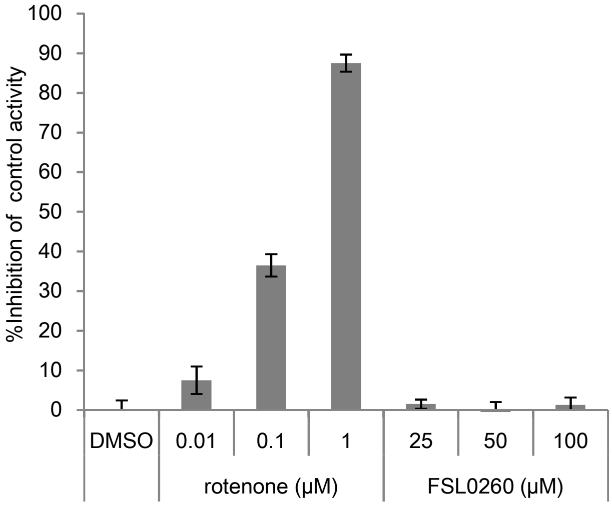

Supplementary figure 5. Inhibition activity of FSL0260 in mammalian mitochondria.
Inhibition of NADH oxidation by FSL0260. NADH oxidation was measured by spectrometry using mitochondria isolated from bovine heart tissue. Rotenone was used as positive control. The experiment was conducted using three biological replicates. Error bars represent the mean ± SE (n = 3).

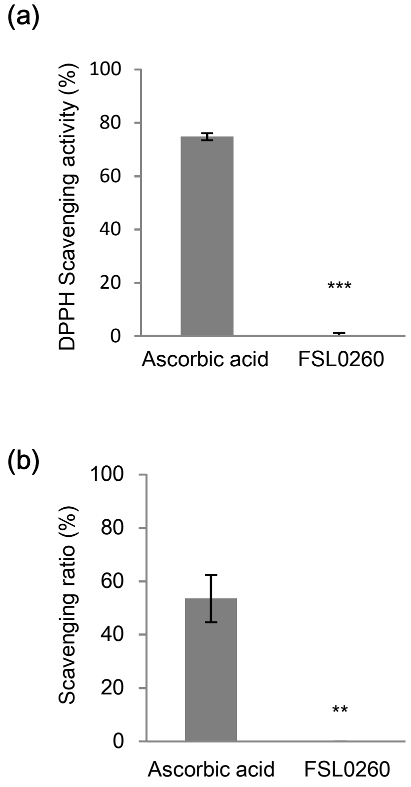

Supplementary figure 6. Radical scavenging activity of FSL0260.
(A) DPPH radical-scavenging activity of 1 mM FSL0260 was measured. 1 mM Ascorbic acid was used as positive control. Error bars represent the mean ± standard deviation (SD). Significance was determined according to Student's t-test. ***P < 0.001.
(B) Superoxide anion radical-scavenging activities of 1 mM FSL0260 was measured. 1 mM Ascorbic acid was used as positive control. Error bars represent the mean ± standard deviation (SD). Significance was determined according to Student's t-test. ***P < 0.01.

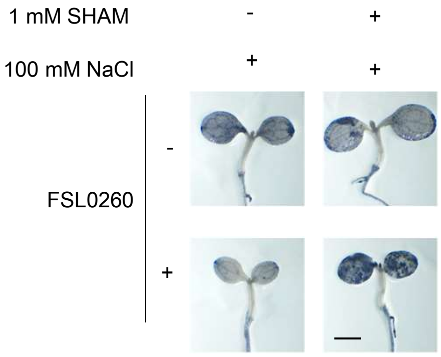

Supplementary figure 7. ROS level of plants treated with both FSL0260 and AOX inhibitor under salt stress**.** NBT staining were used to assess the accumulation of O_2•−. *Arabidopsis thaliana* plants were treated with or without FSL0260 for 24 h, and then treated with NaCl for 6 h in the presence or absence of 1 mM SHAM. Bar = 1 mm. Three biological repeats were performed.

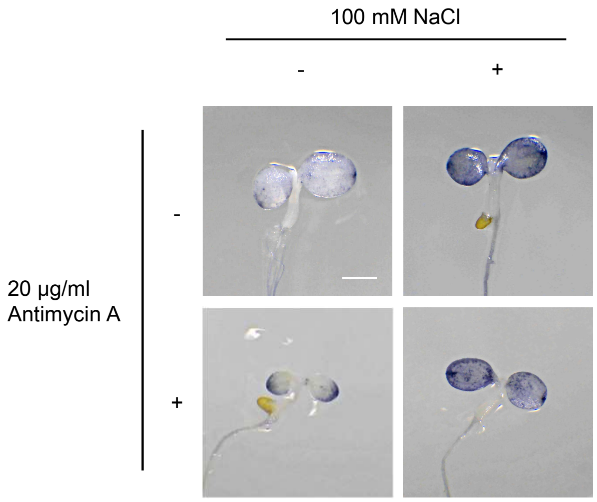

Supplementary figure 8. ROS level of plants treated with antimycin A under salt stress**.** NBT staining were used to assess the accumulation of O_2•−. *Arabidopsis thaliana* plants were treated with or without Antimycin A for 24 h, and then treated with NaCl for 6 h. Bar = 1 mm. Three biological repeats were performed.

Figure 2c

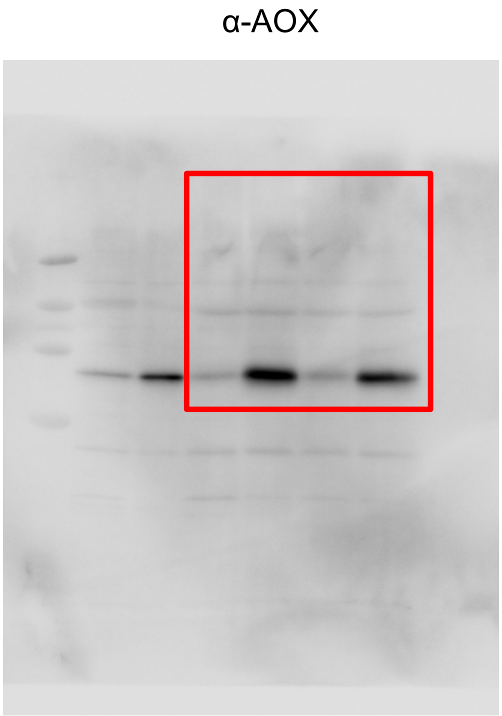

Figure 2d

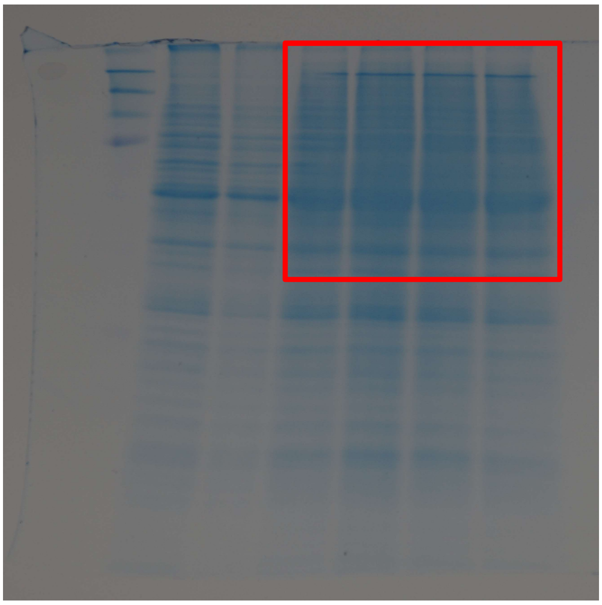

Supplementary figure 9. uncut blots
The red sections mark blot results shown in the indicated figures.

Figure 4c

α-Gamma-CA

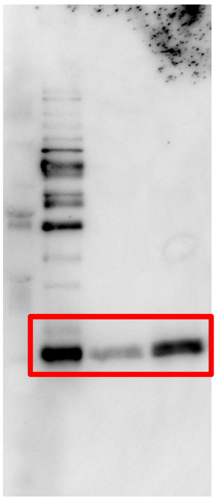

α-COX II

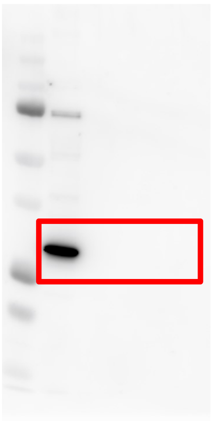

Supplementary figure 10. uncut blots
The red sections mark blot results shown in the indicated figures.

Table S1. Genes up-regulated by FSL0260 treatment.

AGI code[a)]	Gene description	ratio[b)]	p-value	FDR	GO cellular component
AT1G02520	*ATP-BINDING CASSETTE B11 (ABCB11)*	1.358	9.577E-05	0.021	plasma membrane
AT1G03660	*Ankyrin-repeat containing protein*	1.186	0.0001089	0.021	-
AT1G05330	*unknown protein*	1.964	2.558E-06	0.007	cytoplasm
AT1G10170	*NF-X-like 1 (NFXL1)*	1.069	3.489E-06	0.007	nucleus
AT1G23550	*similar to RCD one 2 (SRO2): NAD+ ADP-ribosyltransferase activity*	1.175	7.204E-05	0.020	nucleus
AT1G24095	*Putative thiol-disulphide oxidoreductase DCC*	1.001	9.567E-05	0.021	-
AT1G32870	*NAC domain protein 013 (NAC013)*	2.210	8.761E-06	0.008	nucleus
AT1G72330	*alanine aminotransferase 2 (ALAAT2)*	1.593	0.0001041	0.021	mitochondria
AT1G76600	*poly polymerase*	1.234	4.727E-05	0.019	nucleus
AT2G03130	*Ribosomal protein L12*	3.428	0.0002774	0.032	chloroplast
AT2G03230	*GCK domain-containing protein*	2.777	1.797E-05	0.011	-
AT2G03760	*sulphotransferase 12 (SOT12)*	3.034	1.398E-05	0.010	cytoplasm
AT2G04050	*MATE efflux family protein*	3.558	6.826E-05	0.020	plasma membrane
AT2G04070	*MATE efflux family protein*	2.876	4.209E-05	0.018	plasma membrane
AT2G05420	*TRAF-like family protein*	2.205	9.364E-05	0.021	chloroplast
AT2G14070	*wound-responsive protein-related*	1.701	0.0001022	0.021	-
AT2G18193	*P-loop containing nucleoside triphosphate hydrolases superfamily protein*	2.881	0.0001954	0.028	plasma membrane
AT2G20800	*NAD(P)H dehydrogenase B4 (NDB4)*	2.838	8.592E-05	0.021	mitochondria
AT2G21640	*unknown protein*	3.102	6.553E-05	0.020	mitochondria, nucleus
AT2G28830	*PLANT U-BOX 12 (PUB12)*	3.596	2.102E-05	0.012	nucleus
AT2G32020	*Acyl-CoA N-acyltransferases (NAT) superfamily protein*	2.076	1.357E-05	0.010	cytoplasm

AT2G35480	*unknown protein*	1.885	5.972E-06	0.007	mitochondria
AT2G36790	*UDP-glucosyl transferase 73C6 (UGT73C6)*	2.561	4.344E-05	0.018	chloroplast
AT2G36800	*don-glucosyltransferase 1 (DOGT1)*	2.295	0.0002214	0.029	chloroplast
AT2G38340	*DEHYDRATION RESPONSE ELEMENT-BINDING PROTEIN 19 (DREB19)*	1.295	0.0001265	0.021	nucleus
AT2G38823	*unknown protein*	5.030	5.329E-05	0.020	nucleus
AT2G41730	*unknown protein*	5.077	2.219E-06	0.007	nucleus
AT2G47520	*HYPOXIA RESPONSIVE ERF (ETHYLENE RESPONSE FACTOR) 2 (HRE2)*	4.322	9.798E-05	0.021	nucleus
AT3G01600	*NAC domain containing protein 44 (NAC044)*	1.791	0.0002716	0.032	nucleus
AT3G11020	*DRE/CRT-binding protein 2B (DREB2B)*	1.049	0.0001395	0.023	nucleus
AT3G22370	*alternative oxidase 1A (AOX1A)*	3.330	2.278E-06	0.007	mitochondria
AT3G25250	*AGC2-1*	1.006	0.0006582	0.048	plasma membrane, nucleus
AT3G25573	*unknown protein*	1.414	9.756E-05	0.021	plasma membrane
AT3G45730	*unknown protein*	2.101	0.0001611	0.024	mitochondria
AT3G54520	*unknown protein*	1.739	0.0003733	0.036	nucleus
AT3G58150	*Optic atrophy 3 protein (OPA3)*	3.613	0.000101	0.021	mitochondria
AT3G61630	*cytokinin response factor 6 (CRF6)*	1.858	6.849E-05	0.020	nucleus
AT4G08555	*unknown protein*	3.026	4.817E-06	0.007	-
AT4G12735	*unknown protein*	5.534	2.832E-06	0.007	peroxisome
AT4G15760	*monooxygenase 1 (MO1)*	1.076	0.0004742	0.041	endoplasmic reticulum
AT4G25930	*unknown protein*	3.337	0.0001042	0.021	-
AT4G27580	*phosphatidylinositol transfer SFH5-like protein*	1.796	0.0003491	0.036	cell wall, mitochondria
AT4G37030	*unknown protein*	2.139	0.0002574	0.032	-
AT4G37370	*cytochrome P450, family 8*	2.090	4.222E-05	0.018	endoplasmic reticulum

AT5G09570	*At12CYS-2*	2.510	3.561E-06	0.007	mitochondria
AT5G13210	*unknown protein*	2.319	0.0001722	0.025	chloroplast
AT5G24640	*unknown protein*	5.835	1.594E-07	0.003	nucleus
AT5G40690	*histone-lysine N-methyltransferase trithorax-like protein*	1.149	8.476E-05	0.021	nucleus
AT5G43450	*2-oxoglutarate (2OG) and Fe(II)-dependent oxygenase superfamily protein*	3.178	6.971E-05	0.020	cytoplasm
AT5G52940	*unknown protein*	4.504	6.867E-06	0.007	-
AT5G54100	*Stomatin-like protein 2 (SLP2)*	1.381	0.0002029	0.028	mitochondria
AT5G54420	*unknown protein*	4.550	2.488E-05	0.013	-
AT5G54450	*unknown protein*	5.826	5.846E-06	0.007	-
AT5G54550	*unknown protein*	4.303	1.678E-05	0.011	mitochondria
AT5G54560	*unknown protein*	3.241	3.121E-05	0.016	mitochondria
AT5G55150	*F-box SKIP23-like protein*	2.891	9.138E-05	0.021	nucleus
AT5G62480	*glutathione S-transferase tau 9 (GSTU9)*	1.952	6.128E-05	0.020	cytoplasm
AT5G66480	*unknown protein*	2.231	5.879E-05	0.020	chloroplast

a) AGI code registered in The Arabidopsis Information Resource (TAIR, https://www.arabidopsis.org/).

b) The values represent the $\log_2$ ratio (plants treated with ethanol for 24 h/plants treated with DMSO for 24 h) >1, q<0.05

Supplemental Table 2. Primer list

Name	sequences
NDA1 qPCR F	CTCCGTGAGAGCAAGGAAGG
NDA1 qPCR R	GGCGAAGTGGAGGGGATATG
NDA2 qPCR F	CGAGAGCAAGGACGCAAAAG
NDA2 qPCR R	CAGTAGGCCGAGATTGAGAC
NDB1 qPCR-F	ACTTCATGGAGCAAGTTGGC
NDB1 qPCR-R	ATGGAAGCACAATCGCCAAC
NDB2 qPCR-F	ACTGACTCTCAAAGAGTTCC
NDB2 qPCR-R	CCGATTTGAACTCTTCGATC
NDB3 qPCR F	GCAACATTGCAAGAAAGCAG
NDB3 qPCR R	TTCTTGCTTCCTGGATCAATTT
NDB4 qPCR F	TTGCCAAGTGTCACCAATGG
NDB4 qPCR R	TGCGCATAAGACCACGAATG
NDC1 qPCR F	CAATGGCCGTTCTCTCCTC
NDC1 qPCR R	ACACCCTTGGCCTCTTGTTA
AOX1a qPCR F	GCCATACTTGGATTCGGCTTTC
AOX1a qPCR R	AAGCCCAAAAGCCCATTGAC

Supplemental methods

Synthesis, general techniques

FSL0260 4 was synthesized according to the procedure shown in Scheme 1 from diol 3, which was obtained by reduction of the known ester 2 [1].

Scheme 1. Synthesis of pyridinium salt FSL0260 **4**

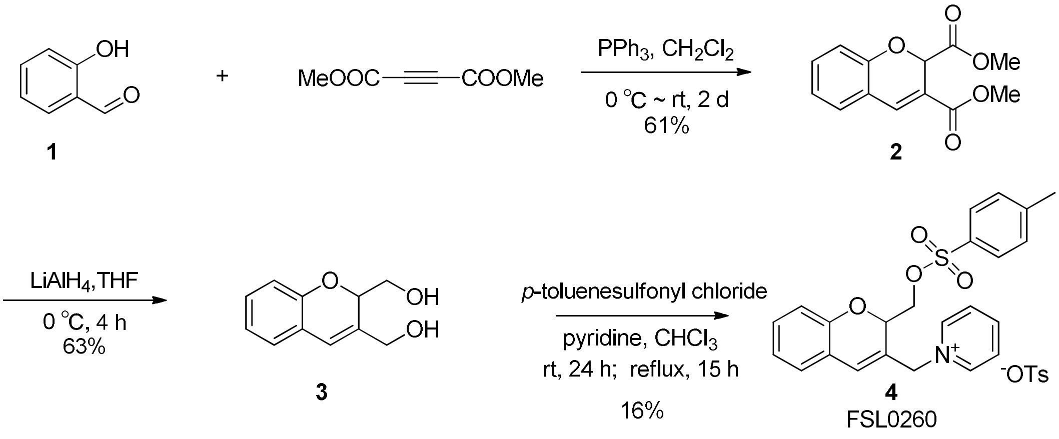

All solvents and reagents were reagent grade. Dehydrated THF was purchased from FUJIFILM Wako Pure Chemical Corporation. Reactions were monitored by thin layer chromatography (TLC) with 0.25 mm E. Merck precoated silica gel plates. Kanto chemical silica gel 60 N (spherical, neutral) (40-50 μm) was used for flash chromatography. ^{1}H NMR spectra were recorded on a JEOL JNM-ECA-500 (500 MHz) spectrometer in $CDCl_3$ with tetramethylsilane (0 ppm) as internal standard and in CD_3OD (3.30 ppm). The following abbreviations were used to explain the multiplicities: s: singlet, d: doublet, t, triplet, q: quartet, m: multiplet, br: broad. ^{13}C NMR spectra were recorded on a JEOL JNM-ECA-500 (125 MHz) spectrometer in $CDCl_3$ as solvent and internal standard (77.0 ppm) and in CD_3OD (49.0 ppm).

(2*H*-Chromene-2,3-diyl)dimethanol (3)

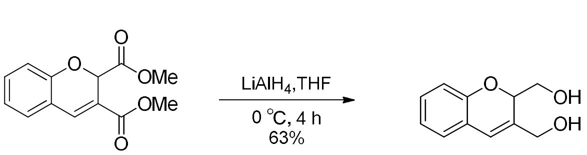

2 3

A solution of **2** (992 mg, 4.0 mmol) in dehydrated THF (10 mL) was added to a stirred suspension of $LiAlH_4$ (455 mg, 12.0 mmol) in THF (10 mL) under N_2 at 0 °C, then the mixture was stirred at 0 °C for 4 h. The reaction mixture was treated by successive dropwise addition of H_2O (0.5 mL), 1*N* NaOH solution (0.5 mL) and H_2O (1.5 ml). The mixture was filtered and the filtrate was concentrated *in vacuo*. The residue

was purified by flash chromatography (*n*-hexane : EtOAc = 1 : 1) to provide **3** as a pale yellow solid (480 mg, 63%).

3 : ^{1}H NMR (500 MHz, $CDCl_3$) δ 3.81 (dd, *J* = 11.5, 4.0 Hz, 2H), 3.87 (dd, *J* = 11.5, 5.5 Hz, 2H), 4.23 (d, *J* = 16.0 Hz, 1H), 4.25 (d, *J* = 16.0 Hz, 1H), 4.94 (dd, *J* = 5.5, 4.0 Hz, 1H), 6.49 (s, 1H), 6.84 (d, *J* = 7.5 Hz, 1H), 6.88 (dd, *J* = 7.5, 7.5 Hz, 1H) 7.00 (dd, *J* = 7.5, 1.5 Hz, 1H), 7.13 (ddd, *J* = 7.5, 7.5, 1.5 Hz, 1H). ^{13}C NMR (125 MHz, $CDCl_3$) δ 63.3, 63.7, 115.8, 121.4, 121.6, 123.0, 126.9, 129.5, 132.8, 152.0.

1-((2-((Tosyloxy)methyl)-2*H*-chromen-3-yl)methyl)pyridin-1-ium 4-methylbenzenesulfonate (4)

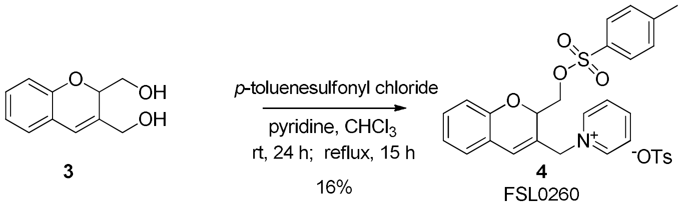

p-Toluenesulfonyl chloride (460 mg, 1.41 mmol) was added to a stirred solution of **3** (90.7 mg, 0.47 mmol) and pyridine (152 µL, 1.88 mmol) in $CHCl_3$ (1 mL) under N_2 at 0 °C. The mixture was warmed to room temperature and stirred for 24 h. Then, the mixture was heated under reflux for 15h. After cooling to room temperature, the mixture was concentrated to afford yellow oil. The oil was diluted with $CHCl_3$ (30 mL) and treated with *p*-toluenesulfonic acid solution (3 mL x 5). The $CHCl_3$ layer was washed with brine, dried over Na_2SO_4, and concentrated to afford a pale yellow solid. The solid was recrystallized from $CHCl_3$ and MeOH to afford **4** as a colorless solid (44.5 mg, 16%). **4** : ^{1}H NMR (500 MHz, CD_3OD) δ 2.35 (s, 3H), 2.42 (s, 3H), 4.09 (dd, *J* = 11.5, 3.0 Hz, 1H), 4.13 (dd, *J* = 11.0, 6.0 Hz, 1H), 5.05 (dd, *J* = 6.0, 3.0 Hz, 1H), 5.38 (d, *J* = 15.5 Hz, 1H), 5.42 (d, *J* = 15.5 Hz, 1H), 6.62 (d, *J* = 8.0 Hz, 1H), 6.72 (s, 1H), 6.90 (ddd, *J* = 7.5, 7.5, 1.0 Hz, 1H), 7.06 (dd, *J* = 7.5, 1.5 Hz, 1H), 7.15 (ddd, *J* = 8.0, 7.5, 1.5 Hz, 1H), 7.21 (d, *J* = 8.0 Hz, 2H), 7.35 (d, *J* = 8.0 Hz, 2H), 7.61 (d, *J* = 8.0 Hz, 2H), 7.69 (d, *J* = 8.0 Hz, 2H), 8.16 (dd, *J* = 7.5, 6.0 Hz, 2H), 8.65 (t, *J* = 7.5 Hz, 1H), 9.04 (d, *J* = 6.0 Hz, 2H). ^{13}C NMR (125 MHz, CD_3OD) δ 21.3, 21.6, 63.4, 69.7, 74.1, 117.3, 121.4, 123.1, 125.3, 127.0, 128.9, 129.0, 129.8, 129.9, 130.8, 131.1, 132.2, 133.8, 141.7, 143.6, 146.2, 146.8, 147.9, 152.8. HRMS(ESI): m/z calcd for $C_{23}H_{22}NO_4S^+[M]^+$ 408.1270, found 408.1259.

Salt stress test using Agar plates

A. thaliana (ecotype Columbia-0) seeds were sterilized and sown in MS medium supplemented with 1% sucrose and 0.8% agar. Seven-day-old plants were transferred to MS medium containing DMSO or 20 µM FSL 0260 for 2 days, and then transferred to MS medium with or without 100 mM NaCl. The root elongations of 15 plants was measured 3 days after the NaCl treatment. The experiment was conducted using three biological replicates.

1,1-Diphenyl-2-picryl-hydrazil (DPPH) free radical scavenging activity

This activity was measured by the procedure described by Blois (1958). The 100 µl of 0.2 mM DPPH mixed with Tris buffer (0.02M, pH 8.0) and sample. Thirty minutes later, the absorbance was measured at 517nm.Percent inhibition was calculated against a control without FSL0260. Ascorbic acid was used as positive control.

Superoxide anion radical scavenging activity

This assay was based on the reduction of nitro blue tetrazolium (NBT) in the presence of nicotinamide adenine dinucleotide (NADH) and phenazine methosulfate (PMS). The 200 µl reaction mixture contained 50 mL of 1M NBT, 150 mL of 1M NADH, Tris buffer (0.02M, pH 8.0) with or without sample. The reaction was started by adding 15 mL of 1M PMS to the mixture and the absorbance change was recorded at 560 nm after 2 minutes. Percent inhibition was calculated against a control without the extract. Ascorbic acid was used as positive control.

Measurement of enzymatic activity of complex II and IV

The enzymatic activity of complex II and complex IV derived from potato tuber were measured according to Huang et al.[2] and Jacoby et al [3], respectively.

in vitro mammalian NADH-CoQ reductase activity test

The enzymatic activity of complex I derived from bovine heart was determined using MitoCheck Complex I Activity Assay Kit (Cat. No: 700930, Cayman Chemical, Ann Arbor, MI, USA) as described previously [4,5].

1 Bayat, M., Imanieh, H. & Hassanzadeh, F. Triphenylphosphine-catalysed one-pot synthesis of γ-butyrolactone derivatives and highly substituted enones via reaction of dimethyl acetylenedicarboxylate and aryl aldehydes. *Tetrahedron Letters* 51, 1873-1875 (2010).

2 Huang, S., Lee, C. P. & Millar, A. H. Activity assay for plant mitochondrial enzymes. *Methods Mol Biol* **1305**, 139-149, doi:10.1007/978-1-4939-2639-8_10 (2015).

3 Jacoby, R., Millar, A. & Taylor, N. Assessment of Respiration in Isolated Plant Mitochondria Using Clark-Type Electrodes. *Methods in Molecular Biology* **1305**, 165-185, doi:10.1007/978-1-4939-2639-8_12 (2015).

4 Robke, L. *et al.* Discovery of the novel autophagy inhibitor aumitin that targets mitochondrial complex I. *Chem Sci* **9**, 3014-3022, doi:10.1039/c7sc05040b (2018).

5 Futamura, Y. *et al.* Bioenergetic and proteomic profiling to screen small molecule inhibitors that target cancer metabolisms. *Biochim Biophys Acta Proteins Proteom* **1867**, 28-37, doi:10.1016/j.bbapap.2018.06.001 (2019).
